# Impact of the duration of the evidence-based medicine use in acute heart failure: A nationwide cohort study

**DOI:** 10.1371/journal.pone.0205440

**Published:** 2018-10-11

**Authors:** Wei-Tsung Wu, Chun-Yuan Chu, Po-Chao Hsu, Wen-Hsien Lee, Ho-Ming Su, Hsueh-Wei Yen, Wen-Chol Voon, Wen-Ter Lai, Sheng-Hsiung Sheu, Tsung-Hsien Lin

**Affiliations:** 1 Division of Cardiology, Department of Internal Medicine, Kaohsiung Medical University Hospital, Kaohsiung, Taiwan; 2 Department of Internal Medicine, Faculty of Medicine, Kaohsiung Medical University, Kaohsiung, Taiwan; Kurume University School of Medicine, JAPAN

## Abstract

**Background:**

Several randomized control trials have established that drugs can decrease the heart failure (HF) rehospitalization in patients with HF. However, limited studies have investigated the duration of medicine use to decrease the rehospitalization period in the real world. Hence, this study aims to investigate whether the evidence-based medicine decreases the HF rehospitalization in different treatment intervals in the clinical practice.

**Method:**

We examined patients admitted with acute HF from the National Health Insurance Research Database in Taiwan. In addition, the major adverse cardiovascular events (MACE) were the composite endpoints of the in-hospital mortality and rehospitalization after 1 year. Furthermore, we analyzed the medicine use to decrease 14 days and 1, 6, and 12 months' HF rehospitalization.

**Results:**

Overall, we examined 11,012 patients. The use of the renin–angiotensin system (RAS) blockers [hazard ratio (HR), 0.58; *P* < 0.01], β-blocker (HR, 0.67; *P* < 0.01), spironolactone (HR, 0.63; *P* < 0.01), and digitalis (HR, 0.67; *P* < 0.01) associated with the lower in-hospital mortality rate. The Cox regression analysis revealed that RAS blocker (HR, 0.86; *P* < 0.01) and β-blocker (HR, 0.71; *P* < 0.01) were independent predictors for MACE. Although RAS blockers declined rehospitalization to 6 months, β-blocker decreased the rehospitalization rate after 1 month use and the benefit persisted till 12 months. Furthermore, digitalis only lowered rehospitalization to 14 days.

**Conclusion:**

This study suggests that the use of evidence-based medicine is associated with lower MACE for patients with HF, and these drugs could play vital roles in different periods to decrease the rehospitalization in the clinical setting.

## Introduction

Lately, heart failure (HF) has become increasingly prevalent worldwide and is associated with considerable morbidities and mortality globally. As HF is a progressive cardiac disease with high mortality risk, it has become the leading cause of hospitalization in recent years. Previously, several evidence-based drugs have been proven in multiple randomized control trials to decrease the rehospitalization of patients with HF. Reportedly, renin–angiotensin system (RAS) blockers can decrease HF rehospitalization both in reduced and preserved HF [1,2]. Although digoxin did not decrease the overall mortality, it decreased the hospitalization rate for worsening HF [3]. In the Treatment of Preserved Cardiac Function Heart Failure with an Aldosterone Antagonist (TOPCAT) trial, the hospitalization rate for HF exhibited a markedly lower incidence in the spironolactone group compared with the placebo group [4]. However, to date, limited studies have investigated the duration of drugs use to decrease the rehospitalization in the clinical setting. Hence, this study aims to assess whether the evidence-based medicine decreases the HF rehospitalization in different treatment intervals in the clinical setting.

## Materials and methods

### Study design and data source

We conducted a cohort study from the National Health Insurance Research Database (NHIRD) containing claims data for patients enrolled in Taiwan's National Health Insurance (NHI) program. The program has provided mandatory universal health insurance since 1995, covers up to 99% of all residents of Taiwan, and contracts with 97% of all medical providers in Taiwan. The NHIRD is a randomly selected dataset of one million NHI claims from the year 2000 registry of all NHI enrollees (NHI 2000). We prospectively followed these patients from January 1, 2000, to December 31, 2008. On the basis of the National Health Research Institutes, no marked differences were observed in age, sex, and healthcare costs between the sampled group and all enrollees in NHI (National Health Insurance Research Database 2000). The database provides comprehensive demographic data, including sex, date of birth, and income level, as well as healthcare data, including the date of admission or discharge, clinical diagnoses (up to five coexisting diagnoses listed on one claims record), medical procedures (up to five diagnostic or therapeutic procedures), expenditures, detailed drug prescriptions, and the number of in-hospital deaths. Since the analyzed data were fully anonymous, the inform consent was waived. This study protocol was also evaluated and approved by the Kaohsiung Medical University Hospital (Kaohsiung, Taiwan) Institutional Review Board (KMUHIRB-EXEMPT(I)-20150057).

### Study cohort

In this study, we enrolled participants who were admitted for acute HF (ICD-9:428) between January 2000 and December 2008. Notably, we selected the first admission date resulted from acute HF if a patient had multiple hospitalization histories. However, we excluded patients if they were diagnosed with malignancy (ICD-9:140–209). Then, we categorized HF cohort patients into two groups, with and without readmission, and wereadjusted according to age, gender and other baseline characteristic (Table 1). In addition, the relevant comorbidities identified were as follows: hypertension (ICD-9:401–404), hyperlipidemia (ICD-9:272.4), cardiac dysrhythmia (ICD-9:427.9), coronary artery disease (ICD-9:414.0, 414.2, 414.8), myocardial infarction (ICD-9:410 Acute MI, 412 Old MI), arthritis (ICD-9:715, 714), diabetes mellitus (ICD-9:250.0, 250.1–250.9), chronic obstructive pulmonary disease (ICD-9:496), depression (ICD-9:296.2, 296.3, 311), chronic kidney disease (ICD-9:585, 585.5), osteoporosis (ICD-9:733.0, 733.01, 733.09), cerebrovascular disease (ICD-9:430–438), asthma (ICD-9:493.0), dementia (ICD-9:290,294.1), substance abuse (ICD-9:303, 304, 305), and schizophrenia (ICD-9:295. 0–295.9) [5]. Overall, we examined 11,012 patients diagnosed with acute HF while admission. Furthermore, we analyzed the difference in clinical outcomes of treatment of evidence-based medicine, including RAS blockers, β-blockers, spironolactone, and digitalis, between HF patients with and without readmission.

**Table 1:** Baseline characteristics.

Variables (*N* = 11,012)	
Age	71.67 ± 14.46
Gender	
Female	5332 (48.42%)
Male	5680 (51.58%)
Income	
0–20,000	9923 (90.11%)
>20,000	1089 (9.89)
Hypertension	7714 (70.05%)
Hyperlipidemia	1357 (12.32%)
Cardiac dysrhythmia	1570 (14.26%)
Ischemic heart disease	5100 (46.31%)
Osteoarthrosis	2823 (25.64%)
Rheumatoid arthritis	240 (2.18%)
Diabetes mellitus	2797 (25.4%)
COPD	2285 (20.75%)
Depression	263 (2.39%)
Chronic kidney disease	1467 (13.32%)
Osteoporosis	432 (3.92%)
Cerebrovascular disease	3276 (29.75%)
Asthma	2108 (19.14%)
Dementia	866 (7.86%)
Substance abuse	80 (0.73%)
Schizophrenia	82 (0.74%)

COPD, chronic obstructive pulmonary disease.

### Study endpoint and definition

The primary endpoint of major adverse cardiovascular events (MACE) was defined as in-hospital mortality or HF readmission at 14, 30, 180, and 365 days. In addition, the definition of evidence-based drugs user is the patient who received treatment for >21 days in 4 weeks.

### Statistical analysis

All data are presented as percentages or means ± standard deviations for continuous variables. We compared categorical variables between groups using the chi-square test. In addition, time-to-event analysis and covariates of risk factors were modeled using Cox's proportional hazards regression model. The follow-up time for survival was started at the date of diagnosis and ended at the date of the development of different outcomes or at the last observation up to December 31, 2008. We computed Kaplan–Meier estimates for the risk among different categories, which were compared by log-rank tests. Furthermore, significant variables in the univariate analysis were selected for the multivariate analysis. We considered *P* < 0.05 as statistically significant. All statistical analyses were performed using the SAS software version 9.2 (SAS Institute, Cary, NC).

## Results

### Baseline characteristics

We analyzed 11,012 patients in this study. Table 1 summarizes the baseline characteristics and comorbidities of enrolled patients with HF. The leading comorbidities were hypertension (70.05%), ischemic heart disease (46.31%), cerebrovascular disease (29.75%), osteoarthrosis (25.64%), and diabetes mellitus (25.5%).

### In-hospital mortality

Age [hazard ratio (HR), 1.03; 95% confidence interval (CI): 1.02–1.04; *P* < 0.0001], use of RAS blockers (HR, 0.58; 95% CI: 0.53–0.63; *P* < 0.01), β-blockers (HR, 0.67; 95% CI: 0.54–0.83; *P* < 0.01), spironolactone (HR, 0.63; 95% CI: 0.53–0.75; *P* < 0.01), and digitalis (HR, 0.67; 95% CI: 0.59–0.76; *P* < 0.01) associated with the lower in-hospital mortality (Table 2).

**Table 2:** The evidence-based medicine and the in-hospital mortality.

	Crude HR	95% CI	*P*	Adjusted HR*	95% CI	*P*
Age	1.03	1.02–1.05	<0.0001	1.03	1.02–1.04	<0.0001
Male	1.03	0.97–1.10	0.3634	1.11	0.95–1.09	0.7089
β-Blockers	0.61	0.49–0.75	<0.0001	0.67	0.54–0.83	0.0003
RAS blockers	0.53	0.49–0.58	<0.0001	0.58	0.53–0.63	<0.0001
Digoxin	0.61	0.54–0.69	<0.0001	0.67	0.59–0.76	<0.0001
Spironolactone	0.56	0.47–0.66	<0.0001	0.63	0.53–0.75	<0.0001

CI, confidence interval; HR, hazard ratio; RAS, renin–angiotensin system.

*Adjusted for age, sex, income category, hypertension, hyperlipidemia, cardiac dysrhythmia, coronary artery disease, myocardial infarction, osteoarthrosis, rheumatoid arthritis, diabetes mellitus, chronic obstructive pulmonary disease, depression, chronic kidney disease, osteoporosis, cerebrovascular disease, asthma, dementia, substance abuse, and schizophrenia.

### MACE

In this study, age (HR, 1.02; 95% CI: 1.01–1.03; *P* < 0.0001), RAS, and β-blockers were independent predictors for MACE. The use of RAS blockers (HR, 0.86; 95% CI: 0.81–0.91; *P* < 0.01) and β-blockers (HR, 0.71; 95% CI: 0.61–0.82; *P* < 0.01) reduced MACE (Table 3).

**Table 3:** Evidence-based medicine and MACE in 365 days.

	Crude HR	95% CI	*P*	Adjusted HR*	95% CI	*P*
Age	1.02	1.01–1.03	<0.0001	1.02	1.01–1.03	<0.0001
Male	1.01	0.96–1.06	0.6611	0.98	0.93–1.03	0.4751
β-Blockers	0.69	0.59–0.79	<0.0001	0.71	0.61–0.82	<0.0001
RAS blockers	0.84	0.79–0.89	<0.0001	0.86	0.81–0.91	<0.0001
Digoxin	0.97	0.90–1.05	0.4239	1.02	0.95–1.11	0.5511
Spironolactone	0.95	0.86–1.05	0.3196	1.00	0.90–1.10	0.9339

CI, confidence interval; HR, hazard ratio; RAS, renin–angiotensin system.

*Adjusted for age, sex, income category, hypertension, hyperlipidemia, cardiac dysrhythmia, coronary artery disease, myocardial infarction, osteoarthrosis, rheumatoid arthritis, diabetes mellitus, chronic obstructive pulmonary disease, depression, chronic kidney disease, osteoporosis, cerebrovascular disease, asthma, dementia, substance abuse, schizophrenia.

RAS blockers associated with decreasing MACE in 14 and 30 days regarding HF rehospitalization (HR, 0.53, 95% CI: 0.41–0.70 and HR, 0.54, 95% CI: 0.45–0.65, respectively, both *P* < 0.01); however, no statistical significance was observed in readmission at 180 and 365 days (Table 4). In addition, the use of β-blockers associated with decreasing HF rehospitalization at 30 days treatment and the benefit persisted up to 12 months (30 days: HR, 0.60, 95% CI: 0.37–0.97; 180 days: HR, 0.76, 95% CI: 0.60–0.98; 365 days: HR, 0.72, 95% CI: 0.58–0.90, all *P* < 0.04), although this benefit was not significant at 14 day readmission (HR, 0.69; *P* = 0.273). Conversely, spironolactone exhibited a neutral effect on 14, 30, and 180 day rehospitalization. Digitalis associated with decreasing 14 day HF readmission (HR, 0.68; 95% CI: 0.46–1.00; *P* = 0.0487); it exerted a neutral effect on the 30 day HF readmission (HR, 0.77; *P* = 0.055), although 6 and 12 month HF readmission increased under the digitalis treatment (HR, 1.28, 95% CI: 1.12–1.45 and HR, 1.32, 95% CI: 1.19–1.48, respectively, both *P* < 0.01). Furthermore, age associated with the increased rehospitalization duration from 180 to 365 days; however, it exhibited a neutral effect on 14 and 30 days. The effect of death was also incorporated for competing risk model and the use of these evidence-based medicine was not affected HF rehospitalization at 365 days (Table 4).

**Table 4:** Evidence-based medicine and rehospitalization in different times.

	Crude HR	95% CI	*P*	Adjusted HR*	95% CI	*P*	Competing model**	95% CI	*P*
**14 days**									
Age	1.00	0.99–1.01	0.9468	1.00	1.00–1.01	0.2842	1.00	0.99–1.01	0.6512
Male	1.24	1.01–1.51	0.036	1.19	0.96–1.48	0.1128	1.25	1.01–1.53	0.0393
β-Blockers	0.65	0.33–1.25	0.1942	0.69	0.36–1.34	0.2731	0.69	0.36–1.34	0.2726
RAS blockers	0.56	0.43–0.73	<0.0001	0.53	0.41–0.70	<0.0001	0.54	0.41–0.70	<0.0001
Digoxin	0.68	0.46–1.00	0.0471	0.68	0.46–1.00	0.0487	0.67	0.46–1.00	0.0473
Spironolactone	0.82	0.52–1.29	0.3931	0.79	0.50–1.24	0.2981	0.79	0.50–1.24	0.3062
**30 days**									
Age	1.00	1.00–1.01	0.6777	1.00	1.00–1.01	0.1583	1.00	1.00–1.01	0.3476
Male	1.17	1.02–1.34	0.0289	1.11	0.85–1.29	0.1824	1.14	0.99–1.32	0.0700
β-Blockers	0.61	0.38–0.97	0.0355	0.60	0.37–0.97	0.0353	0.60	0.37–0.97	0.0386
RAS blockers	0.58	0.48–0.69	<0.0001	0.54	0.45–0.65	<0.0001	0.55	0.45–0.66	<0.0001
Digoxin	0.74	0.57–0.95	0.0204	0.77	0.59–1.01	0.0555	0.77	0.60–1.01	0.0573
Spironolactone	0.91	0.68–1.23	0.5367	0.92	0.68–1.24	0.5844	0.92	0.68–1.25	0.5992
**180 days**									
Age	1.01	1.00–1.03	<0.0001	1.01	1.00–1.02	<0.0001	1.01	1.00–1.01	0.0008
Male	1.09	1.01–1.18	0.0336	1.07	0.98–1.17	0.1365	1.06	0.98–1.15	0.1662
β-Blockers	0.76	0.60–0.97	0.028	0.76	0.60–0.98	0.0330	0.79	0.62–1.01	0.0608
RAS blockers	0.91	0.83–1.00	0.0445	0.89	0.81–0.98	0.0217	0.91	0.83–1.00	0.0525
Digoxin	1.20	1.06–1.36	0.0047	1.28	1.12–1.45	0.0002	1.25	1.10–1.43	0.0005
Spironolactone	1.09	0.93–1.28	0.3034	1.12	0.95–1.31	0.1943	1.11	0.95–1.31	0.1970
**365 days**									
Age	1.01	1.00–1.03	<0.0001	1.01	1.00–1.02	<0.0001	1.01	1.00–1.01	0.0008
Male	1.09	1.01–1.17	0.0193	1.07	0.99–1.16	0.0856	1.06	0.98–1.14	0.1469
β-Blockers	0.75	0.60–0.92	0.0071	0.72	0.58–0.90	0.0037	0.75	0.61–0.94	0.0116
RAS blockers	1.06	0.98–1.14	0.1756	1.02	0.94–1.11	0.5970	1.04	0.95–1.12	0.4142
Digoxin	1.25	1.12–1.39	<0.0001	1.32	1.19–1.48	<0.0001	1.28	1.14–1.43	<0.0001
Spironolactone	1.19	1.04–1.37	0.0114	1.20	1.04–1.38	0.0102	1.19	1.03–1.36	0.0153

CI, confidence interval; HR, hazard ratio; RAS, renin–angiotensin system.

*Adjusted for age, sex, income category, hypertension, hyperlipidemia, cardiac dysrhythmia, coronary artery disease, myocardial infarction, osteoarthrosis, rheumatoid arthritis, diabetes mellitus, chronic obstructive pulmonary disease, depression, chronic kidney disease, osteoporosis, cerebrovascular disease, asthma, dementia, substance abuse, and schizophrenia.

** Incorporated death for competing model.

## Discussion

The four major findings of this study are as follows: (a) the use of RAS and β-blockers were associated with the lower in-hospital mortality and rehospitalization after 1 year; (b) the use of RAS blockers, spironolactone, β-blockers, and digitalis were associated with the lower in-hospital mortality; (c) the use RAS and β-blockers were associated the lower rehospitalization until 6 months; and (d) digitalis was associated with lower rehospitalization after the 6 month use.

The prognosis of long-term patients with chronic HF is profoundly poor and exhibits a high mortality and rehospitalization rate. The evidence-based medical therapy, including RAS blockers, β-blockers, and spironolactone can decrease the long-term mortality and rehospitalization. Reportedly, the use of enalapril in the SOLVD trial investigating patients with chronic HF with low ejection fraction (EF) revealed that angiotensin-converting enzyme inhibitor (ACEI) therapy markedly decreased the mortality and hospitalization rate over a period of 48 months [1]. In the Valsartan Heart Failure Trial, valsartan markedly decreased the combined endpoint of mortality and morbidity, especially in the subgroup without ACEI background therapy (44% risk reduction) [6]. This cohort study presented similar results that the RAS blocker treatment can decrease MACE, including the in-hospital mortality and HF rehospitalization rate, after 6 month treatment. Previous studies, including CIBIS-II (1.3 years), MERIT-HF (1 year), and COPERNICUS (316 days), reported that β-blockers could decrease the mortality and HF hospitalization rate in average 1 year follow-up [7–9]. Likewise, this study demonstrated that β-blocker therapy reduced MACE in 1 year follow-up. Furthermore, these trials concluded that β-blockers and RAS blockers are cornerstones of the evidence-based medicine for the HF treatment that could decrease the long-term in-hospital mortality and rehospitalization rate, and the time of β-blocker therapy should be >12 months.

The evidence-based medical therapy comprising RAS blockers, β-blockers, and spironolactone could effectively decrease mortality in patients with HF. In addition, mineralocorticoids play a vital role in the pathophysiology of HF. Moreover, the treatment with spironolactone could decrease both HF rehospitalization and mortality in HF with decreased EF patient according to the Randomized Aldactone Evaluation Study [10]. Eplerenone, one of the mineralocorticoid antagonists, could decrease both the risk of mortality and hospitalization among patients with systolic HF and mild symptoms [11]. Similarly, this study revealed that the in-hospital mortality rate decreased after the spironolactone treatment, as well as RAS and β-blockers.

The literature review in this study revealed that the evidence-based medicine, including β-blockers, RAS blockers, and spironolactone, could also reduce rehospitalization. In this study, the use of β-blocker therapy for >30 days decreased HF rehospitalization, and this benefit was sustained for 12 months. In addition, RAS blockers could decrease rehospitalization until the 6 month treatment. In the TOPCAT trial, although the spironolactone treatment in patients with HF with preserved EF did not decrease mortality, the hospitalization rate for HF was lower in the spironolactone group. In addition, the side effect of the elevated serum creatinine level and the hyperkalemia rate was reported in the spironolactone group [4]. In this study, the spironolactone exhibits a neutral effect on HF rehospitalization between 14 and 180 day treatment. However, the different results of spironolactone between studies could be attributed to the different severity of patients and comorbidities of the enrolled population.

Reportedly, in geriatric patients with chronic HF, the digitalis reduced hospitalizations because of HF at low serum digoxin concentrations [12,13]. In addition, the Dig Trail revealed the reduction rate of HF hospitalization and HF worsening; however, the overall mortality was neutral [3]. This study demonstrates that the digitalis treatment decreased HF readmission in 14 day therapy but increased the HF readmission rate after 6 months use. Interestingly, the digitalis therapy in this study also decreased the in-hospital mortality rate in patients with HF, implying that the short-term digitalis therapy could be beneficial but should be cautiously used in long-term therapy.

### Limitations

This study has several limitations. First, the population included patients admitted for HF with both preserved and reduced EF, which cannot be distinguished from NHIRD. Second, the study cohort only included patients who had ever admitted because of HF. Third, the sample size is limited; however, it actually could represent patients with HF actually hospitalized in Taiwan. Fourth, it was not available for drugs doses and blood levels, which might affect the drug effect and cardiovascular outcomes. Fifth, we were unable to control the clinical severity by controlling comorbidities alone that might affect the hospital mortality rate. Finally, this observational cohort study can only provide association rather than causation.

## Conclusions

The evidence-based medicine including RAS and β-blockers could be associated with lower MACE for patients with HF. Both RAS and β-blockers could benefit patients by reducing HF readmission and in-hospital mortality rates until 12 months. Furthermore, RAS blockers, β-blockers, spironolactone, and digitalis might play vital roles in a different time to decrease rehospitalization in the clinical setting.
